# OmicsTIDE: interactive exploration of trends in multi-omics data

**DOI:** 10.1093/bioadv/vbac093

**Published:** 2023-01-20

**Authors:** Theresa A Harbig, Julian Fratte, Michael Krone, Kay Nieselt

**Affiliations:** Institute for Bioinformatics and Medical Informatics, University of Tuebingen, Tuebingen 72076, Germany; Institute for Bioinformatics and Medical Informatics, University of Tuebingen, Tuebingen 72076, Germany; Institute for Bioinformatics and Medical Informatics, University of Tuebingen, Tuebingen 72076, Germany; Institute for Bioinformatics and Medical Informatics, University of Tuebingen, Tuebingen 72076, Germany

## Abstract

**Motivation:**

The increasing amount of data produced by omics technologies has enabled researchers to study phenomena across multiple omics layers. Besides data-driven analysis strategies, interactive visualization tools have been developed for a more transparent analysis. However, most state-of-the-art tools do not reconstruct the impact of a single omics layer on the integration result.

**Results:**

We developed a data classification scheme focusing on different aspects of multi-omics datasets for a systemic understanding. Based on this classification, we developed the Omics Trend-comparing Interactive Data Explorer (OmicsTIDE), an interactive visualization tool for the comparison of gene-based quantitative omics data. The tool consists of a computational part that clusters omics datasets to determine trends and an interactive visualization. The trends are visualized as profile plots and are connected by a Sankey diagram that allows for an interactive pairwise trend comparison to discover concordant and discordant trends. Moreover, large-scale omics datasets are broken down into small subsets that can be analyzed functionally using Gene Ontology enrichment within few analysis steps. We demonstrate the interactive analysis using OmicsTIDE with two case studies focusing on different experimental designs.

**Availability and implementation:**

OmicsTIDE is a web tool available via http://omicstide-tuevis.cs.uni-tuebingen.de/.

**Supplementary information:**

Supplementary data are available at *Bioinformatics Advances* online.

## 1 Introduction

With the advent of high-throughput technologies, it has become affordable to comprehensively study all entities in one omics layer of a sample, e.g. all genes, transcripts, proteins or metabolites. While studying single omics layers already requires sophisticated method, analyzing multiple omics layers across several experimental conditions adds a whole new level of complexity. Therefore, the demand for methods that integrate and visualize multiple omics datasets has been steadily increasing over the past decades.

While a data-driven integration can derive interesting relations between different omics layers, it often is perceived as a black box. For instance, the impact of single genes or groups of genes on the integration is not always evident. To overcome this limitation of purely data-driven methods, different approaches have been developed (Hernández-de Diego *et al.*, 2018; Kuo *et al.*, 2013). Many of these approaches reduce the complexity of the datasets by classifying single genes into different categories based on their ‘behavior’. For example, in a dataset that deals with two conditions, a gene could be classified as being *up-regulated* in one condition with respect to the other condition. The situation becomes more complex when more than two conditions are observed. This requires the application of clustering methods to obtain representative *trends* for sets of genes. Here, we define a *trend* in omics abundance data as a set of omics-entities that follow a distinct trajectory across at least two conditions.

To provide a tool that overcomes the current limitations in the omics visualization field, we first devised a general classification system for omics data that categorizes the data to be analyzed and compared with respect to their data type as well as experimental design. A detailed categorization preceding an integrated omics analysis will help to choose a suitable analysis approach. This classification builds the framework for the *Omics Trend-comparing Interactive Data Explorer* (OmicsTIDE), a tool that creates a connection between the single genes and the trends derived from gene-based quantitative multi-omics datasets including, for example, transcriptomic and proteomic data.

The comparison of trends found in two omics layers is the central concept of OmicsTIDE, which visualizes trends as profile plots, also known as parallel coordinate plots (Inselberg, 1985), and compares trends between two datasets using a Sankey diagram (Lex *et al.*, 2012). OmicsTIDE aims to identify the same trends in both datasets, therefore, genes are further grouped into whether they follow concordant (i.e. the same) or discordant (i.e. different) trends. Moreover, the tool breaks down large-scale datasets into small subsets within a few steps based on selecting groups of genes in the Sankey diagram. These subsets can be functionally analyzed using Gene Ontology enrichment analysis. By allowing several pairwise comparisons within a single analysis, OmicsTIDE combines insights from different pairwise comparisons into one large analysis. We demonstrate the effectiveness of OmicsTIDE in two case studies with different experimental designs.

## 2 Related work

For this article, we define a multi-omics tool as a tool that integrates and visualizes data of two or more omics layers concurrently. In this related work section, we therefore focus on tools that analyze different omics data in a combined instead of a separated or sequential manner.

The most straightforward way of visualizing multi-omics data is mapping them directly to a genome sequence or a pathway. Any kind of omics data that can be mapped to a genome sequence can be represented in genome coordinate-based visualizations such as genome browsers (Nusrat *et al.*, 2019). With tracks stacked upon each other, various omics layers can be displayed simultaneously. Similarly, omics data can be mapped to a pathway ID in a node-link diagram, where genes, proteins and metabolites can be shown simultaneously (Luo and Brouwer, 2013). While genome browsers and pathway maps intuitively visualize multi-omics data, they usually show only a small window of the genome or a single pathway of interest and are limited to a small number of conditions that can be displayed simultaneously.

Moreover, various computational methods have been developed for the integration of multi-omics data, as reviewed by Bersanelli *et al.* (2016) and Huang *et al.* (2017). Often omics data are clustered using advanced clustering approaches (Rappoport and Shamir, 2018; Tini *et al.*, 2019), which can be divided into *early integration* and *late integration* approaches. While early integration approaches first concatenate the data of different omics layers and then cluster the merged data, late integration methods find patterns in the features of each layer separately, which can be combined as input for a regression or classification (Sharifi-Noghabi *et al.*, 2019). For early integration, data can either be concatenated by omics-features (rows) or conditions (columns). OmicsTIDE applies an early integration approach by concatenating two omics datasets by condition and clustering the concatenated matrix.

Commonly, the results of the integration methods are visualized in node-link diagrams or in trend visualizations, such as heatmaps and profile plots. The tool 3Omics clusters up to three different omics layers, e.g. transcriptomics, proteomics and metabolomics data hierarchically and visualizes the results as a clustered heatmap (Kuo *et al.*, 2013). Alternatively, it creates correlation networks as node-link diagrams. A similar heatmap visualization has been implemented in the tool multiSLIDE, which combines two heatmaps side-by-side comparing transcriptomics and proteomics data (Ghosh, 2020). While heatmaps represent one of the most commonly used approaches for visualizing abundance data, they can become huge when analyzing a large number of genes. Due to their size and because of the usage of the color encoding, trends may become difficult to determine (Gehlenborg and Wong, 2012).

Paintomics follows an alternative integration approach for multiple omics layers by associating the omics-features, such as genes, proteins and metabolites with their respective KEGG pathways and conducting pathway enrichment (Hernández-de Diego *et al.*, 2018). Each pathway can be analyzed in detail where the major trends of the associated features in different omics layers are displayed. However, it does not show to what degree the single omics-features contribute to the final trend and trends cannot directly be compared between pathways.

For trend comparison different strategies have been developed. For instance, an approach to visualize and compare trends was demonstrated in a study on the comparison of the transcriptomes of *Arabidopsis thaliana* and *Zea mays* (Vercruysse *et al.*, 2020), where trends in orthologous genes in leaf development were determined and compared. For the visualization of trends, the authors use profile plots, which are compared between the organisms using a table showing the orthologous genes overlapping between the trends. This approach provides a good overview of the trends in the two datasets. However, for the hierarchical clustering for categorization into discrete trends, clusters have to be separated manually. Moreover, as clustering was done independently for each dataset, there is no inherent concept for trends having the same or different trajectories in the datasets. Thus, genes cannot be classified as following the same or different behaviors.

Despite the fact that many tools have been developed to integrate multi-omics data, approaches integrating the data computationally, while keeping the integration process transparent using an exploratory visualization are rare. Overcoming this limitation was the main motivation for the development of OmicsTIDE.

## 3 Classification of omics data

**Fig. 1.**
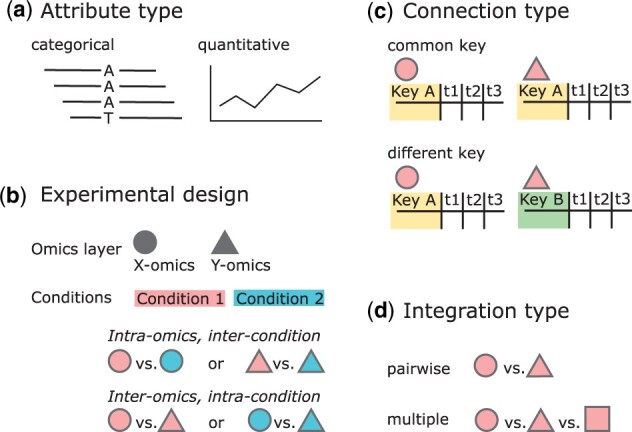
Omics data can be classified in different ways. (**a**) The *attributes* of omics features are either of categorical (e.g. mutations) or quantitative (e.g. transcript/protein levels) type. (**b**) Omics data analysis can also be classified by the *experimental design*. The analysis typically includes either the comparison of different conditions in the same omics layers (*inter-condition* and *intra-omics*) or the comparison of data from different omics layers within the same condition (*intra-condition* and *inter-omics*). (**c**) The combined analysis of omics datasets can be classified by whether the datasets can be joined by a common *key attribute* or not. (**d**) The *integration* of different omics layers can either be done in a pairwise fashion or by directly comparing multiple layers at once

To identify the requirements for a novel multi-omics visualization tool and to create an abstract representation of the data, we developed a classification scheme. This classification builds the requirement framework for OmicsTIDE. First, omics data can be classified by the *attribute type*, which can either be categorical, such as the different bases of SNPs in genomics research, or quantitative, such as expression levels of genes, proteins or metabolites (Fig. 1a).

Secondly, comparative omics experiments can be classified by experimental design, which depends on the research goals (Fig. 1b). Experiments are often performed within an omics layer (*intra-omics*) and between different conditions (*inter-condition*). Alternatively, omics experiments can include multiple omics layers (*inter-omics*) studying the same biological condition (*intra-condition*). In the case studies section, we show a use case with an *inter-omics* and *intra-omics* experimental design. The design is applied to find differences in the transcriptomes of two strains under the effect of phosphate depletion (*intra-omics, inter-condition*), and to study how these differences are reflected in the proteome (*inter-omics, intra-condition*) (Sulheim *et al.*, 2020).

When the *inter-omics* approach is chosen as experimental design, the connection between datasets can be created based on common keys with which the datasets can be combined or compared (Fig. 1c). If the datasets do not share keys, a direct comparison cannot be conducted.

For *inter-omics* experimental designs, the decision on the number of omics layers determines the subsequent downstream analysis steps (Fig. 1d). To study a given biological question, it might be sufficient to compare two omics layers. More complex questions might require more than two omics studies (*multi-omics*) for a more powerful analysis to find specific patterns in the integrated datasets.

## 4 Methods

Based on the classification scheme and our survey of related work, we developed four goals for the development of OmicsTIDE:

1. *Interpretability:* Provide a balance between sophisticated integration and interpretability for users.
2. *Applicability:* Focus on data that is widely available. Proteomics and Transcriptomics data are some of the most commonly produced data types.
3. *Overview-Detail:* Provide an overview of the integration, while also allowing detailed analysis of small subsets of the data or even single omics-features.
4. *Functional analysis:* Summarize subsets of omics features functionally to gain insights about the underlying biological context.

OmicsTIDE employs pairwise integration of two omics layers (*inter-omics*, *intra-condition*) and the comparison of two datasets within an omics layer (*intra-omics*, *inter-condition*) using a simple concept of clustering the data into trends of omics features following the same trajectory (Goal 1). OmicsTIDE only requires data from one or two omics layers to produce meaningful results (Goal 2). The tools offer a detailed analysis of omics-features of interest identified in the overview visualization (Goal 3). Groups of omics-features can be analyzed functionally using GO-term enrichment (Goal 4).

OmicsTIDE computes and visualizes trends for two-dimensional experimental designs. The first dimension is represented by the datasets that are compared, which can be from one or two different omics layers. The second dimension is represented by conditions that need to be consistent across datasets, such as time points or environmental conditions.

The central idea of the visualization approach is to compare trends occurring in two omics datasets using a Sankey diagram, which is a graphical representation of flows between sets. The trends of the different datasets are visualized adjacent to the *nodes* of the Sankey diagram. The height of the nodes encodes for the number of genes found in the trends, while the thickness of the bands (*links*) between the nodes encodes for the number of genes that either show the same trends (concordant trends) or different trends (discordant trends) in the two datasets.

**Fig. 2.**
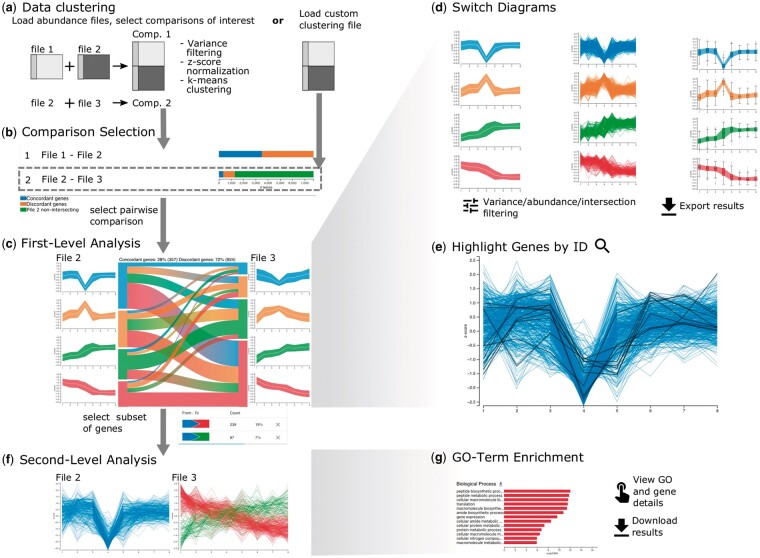
Basic workflow using OmicsTIDE. (**a**) Either multiple abundance files or a single custom trend-comparison file can be uploaded. (**b**) An overview of all conducted pairwise trend comparisons is shown as horizontal stacked bar chart showing the count of genes either being found in both compared files (intersecting) or only in one of the two files (non-intersecting). The number of intersecting genes in the bar chart is further categorized by either following a concordant or discordant trend in the two compared files. (**c**) After selecting a pairwise comparison in the overview visualization the data can be analyzed in the first-level analysis, consisting of a Sankey diagram comparing trends in both abundance files. (**d**) Users can switch between different trend diagrams, (**e**) highlight genes using gene IDs and filter data by abundance and variance. (**f**) For a more detailed analysis subsets of genes can be analyzed in the second-level analysis in detailed profile plots showing the expression of single genes (*y*-axis) across conditions (*x*-axis). Moreover, second-level analysis includes (**g**) viewing the NCBI entries of single genes and GO term enrichment analysis in a bar chart. The *x*-axis corresponds to the –log_10_ (FDR) (false discovery rate) values and the *y*-axis corresponds to the significantly enriched GO-tems (FDR < 0.05). The color of the bars encodes for the term being overrepresented or underrepresented

Datasets are compared in three major steps referred to as *comparison selection*, *first-level analysis* and *second-level analysis* (Fig. 2). The separation of the analysis is reflected in the dynamic tab-based design of OmicsTIDE, with which new tabs corresponding to the respective analysis steps can be added. With this design, choices made in any tab can be reviewed, refined or removed.

During all steps of the analysis intermediate results can be exported in CSV format for downstream analysis with other tools. For easy sharing of the visualizations, they can be exported in PNG or PDF format.

### 4.1 Data loading and comparison selection

OmicsTIDE offers two distinct data input options in form of abundance files or a custom clustering file (Fig. 2a). Users can load multiple abundance files that are compared in a pairwise fashion and clustered by OmicsTIDE to obtain trends. Each abundance file containes genes (rows), conditions (columns) and normalized abundance (cells). After choosing abundance files, users can choose to restrict the analysis to variant genes by removing genes based on the percentile range of their variances across different conditions. This reduces the formation of trends that are influenced by low-variance genes. Moreover, if more than two datasets are to be analyzed, the pairwise comparisons of interest can be selected. For fast exploration of the data OmicsTIDE uses k-means. For this, the number of trends to be derived from the data has to be chosen in a range between 2 and 10. To make all datasets comparable, OmicsTIDE applies *z*-score normalization to each dataset prior to clustering. To guarantee flexibility in the choice of clustering algorithms, users can upload their own clustering results for a pairwise trend comparison.

For each pairwise combination, OmicsTIDE conducts two separate trend comparisons: One for the genes found in both datasets (intersecting genes) and one for the genes found only in one of the two datasets (non-intersecting genes). While non-intersecting genes are clustered separately for each dataset, for the intersecting genes, OmicsTIDE makes use of an *early integration* approach by first combining the two datasets using the shared conditions and then clustering the combined matrix with k-means++ (Arthur and Vassilvitskii, 2006). Therefore, each gene is associated with two clusters, one for each dataset. With this approach, the genes can easily be classified as following concordant or discordant trends, which represents an intuitive concept for users.

An overview visualization helps users choose a comparison for the trend exploration in the first-level analysis (Fig. 2b). The comparisons are visualized using stacked bar charts showing concordant, discordant, intersecting and non-intersecting genes and are thus providing a useful rationale to select a comparison of interest.

### 4.2 First-level analysis: trend exploration

The first-level analysis tab provides a detailed visualization of the selected trend comparison of intersecting genes in a Sankey diagram together with profile plots (Fig. 2c, Supplementary Fig. S1), as well as a sidebar containing controls for interactive features (Supplementary Fig. S1). The Sankey diagrams shows the size of the gene sets corresponding to the trends (nodes) and how many genes are contained in the trend intersections, i.e. shared between the trends of the two datasets (links). The trends, nodes and links are colored using a set of categorical colors. A color-gradient is used for the bands transitioning between the colors of the connected nodes. The gradient is inverted and shows the color of the left node on the right side and vice versa to simplify the identification of trends in one dataset connected to a single trend in the other dataset by looking at the corresponding node. A summary of the comparison is displayed at the top of the visualization, showing the number and percentage of concordant and discordant genes.

By default, the trends are visualized using *centroid profile plots*, which provide an overview by showing the centroid line as well as the standard deviation of the trend as a band. Alternatively, using the controls sidebar users can choose profile plots, where each gene is plotted as a line for a more detailed view on the composition of each trend (Fig. 2d). This visualization is more suitable for a low number of genes since the visualization of a large number of gene profiles may result in overplotting. As a third option, the user can study the abundance variation per condition within a trend in more detail using box plots. In addition to the analysis of intersecting genes, the trend visualizations are used for analyzing non-intersecting genes. Since non-intersecting genes do not share identifiers, they are not connected with a Sankey diagram but only show trend visualizations and horizontal bar charts to show sizes of the gene sets corresponding to the trends (Supplementary Fig. S2).

The nodes and links in the Sankey diagram can be hovered to study the single trends between the two datasets in more detail. When a node is hovered, all connected links are highlighted by reducing the opacity of other elements (*focus-on-hover* strategy). Hovering over elements in the visualization updates the detail diagrams accordingly. This update is facilitated via an animated transition to visually link the hovering and the data update. Moreover, all concordant or discordant intersections can be highlighted by hovering over the concordance/discordance summary.

Users can check their own hypotheses about gene sets of interest, such as genes from specific pathways, and analyze their behavior across trends and datasets by highlighting genes of interest by their gene IDs (Fig. 2e). Users can directly type in one or more gene IDs into a text field or upload a text file with gene IDs. The profiles of the genes in the diagram corresponding to the given IDs in the query are marked in black.

To study the effects of the variance or abundance levels of genes on the trends, OmicsTIDE can dynamically filter data by the percentile ranges of the variance or the median abundance of the genes during first-level analysis. The variance and the median abundance and their respective percentiles are calculated prior to *z*-score normalization. The variance filtering in the first-level analysis can be applied as an alternative or in addition of the variance filtering provided when loading the data. In contrast to the variance filtering before loading the data, which is considered a pre-processing step, the filtering in the first-level analysis allows users to explore different ranges of variances quickly. In addition, users can filter intersections by size to remove small intersections from the visualization and to thus reduce visual clutter.

### 4.3 Second-level analysis: detailed trend analysis

Sets of genes corresponding to trends or the intersection of trends can be analyzed in detail to find, for example, enriched functions, thus implementing our fourth goal. OmicsTIDE allows users to select either links or nodes in the visualization to extract subsets of genes. A table placed in the controls side bar shows the source node and the target node of each selected link as well as the number and percentage of the corresponding genes (Fig. 2c, bottom). Thereby, users can compare the actual numbers of genes corresponding to a link.

Selected genes can then be analyzed in detail in the second-level analysis (Fig. 2f). Users can study gene sets on the single-gene level by hovering the single-gene profiles and accessing information of an individual gene by clicking and being redirected to the corresponding NCBI entry. Furthermore, the gene subsets can be analyzed in a gene ontology (GO) context (Fig. 2g). Thereby, users can find GO terms that are enriched in in the selected subset and form hypotheses about the regulatory processes causing the patterns. The PantherDB API is used to perform GO enrichment for the three main GO categories *molecular function*, *biological process* and *cellular component* using *Fisher’s exact test* and a multiple test correction with false discovery rate (FDR) (Mi *et al.*, 2021). Users can either choose to use the whole genome as a background for the enrichment, or only the genes contained in the current first-level analysis, as certain classes of proteins can, for example, be underrepresented in proteomics data. The results are visualized as horizontal bar charts where length encodes for the negative logarithm of the *FDR* and color encodes for the term being overrepresented or underrepresented to allow users to quickly identify the most significant results. Hovering the single bars will show a tool tip with more detailed information on the corresponding GO term. Clicking on the bar will open a tab with further information about the GO term on Amigo (http://amigo.geneontology.org/).

### 4.4 Implementation

OmicsTIDE is a web-based client–server application that uses *Python* for complex computations, such as the trend determination via clustering in the back-end and Flask for communication with the front-end (Grinberg, 2018). The libraries React (https://reactjs.org/) and Mobx (https://mobx.js.org/) are used for the application structure of the front-end and state-management. The JavaScript library D3.js is used for creating the visualizations and animation (Bostock *et al.*, 2011). The application styles are created using Material-ui (https://mui.com/). The source code of OmicsTIDE is available at https://github.com/Integrative-Transcriptomics/OmicsTIDE2.0.

## 5 Case studies

To demonstrate the applicability of the pairwise trend comparison approach in OmicsTIDE, we conducted two case studies. In the first case study, we show how the combined analysis of transcriptomics and proteomics data can be used to extract biologically relevant concordant as well as discordant trends with few clicks only. The second case study combines two pairwise trend comparisons to extract information from both, different experimental conditions and different omics layers to demonstrate the synergy that can be achieved by OmicsTIDE.

### 5.1 Blood cell differentiation in bone marrow

Neutrophils are an essential part of the human immune system. They are differentiated in the bone marrow and released to the bloodstream. The regulation of the neutrophil differentiation is subject of the first case study, examining granulopoiesis *in vivo* (Hoogendijk *et al.*, 2019). The experimental design uses both transcriptome and proteome data from the five differentiation stages (pro)myelocytes (PMs), metamyelocytes (MMs), immature neutrophils with band-shaped nucleus (BN), mature neutrophils with segmented nucleus (SNs) and the actual peripheral mature neutrophils (PMNs). Here, we show how OmicsTIDE can be used to efficiently reproduce the findings made by Hoogendijk *et al.* (2019) by exploring the trends between the two omics layers. The data was taken from the supplementary material that contained quantified transcripts and proteins in the form of *Fragments Per Kilobase Million* (FPKM) and imputed log_2_*Label Free Quantification* (LFQ) measures, respectively. The data included four replicates for transcripts and three replicates for proteins for each of the five conditions. The analysis was performed on the mean values of all biological replicates for each condition.

**Fig. 3.**
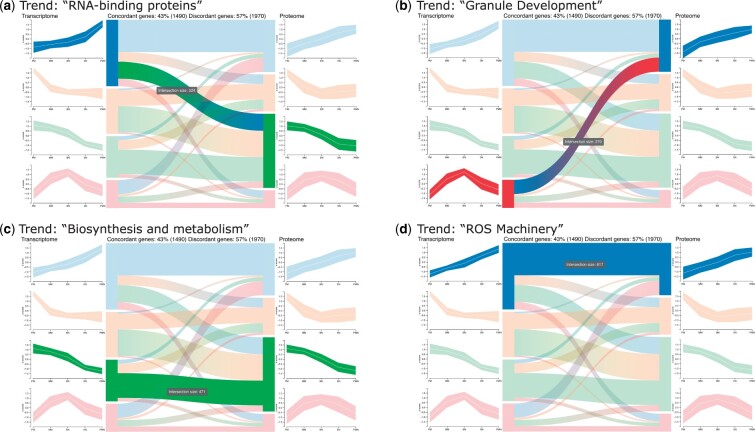
First-level analysis: Interactively studying the trend comparison between the transcriptome and proteome during blood cell differentation (Hoogendijk *et al.*, 2019) by hovering reveals several concordant and discordant trends. (**a–d**) Four trends found by OmicsTIDE were associated with functionally annotated modules found in the study. The tooltip displays the number of contained genes

To explore the trends shown by the transcriptome and the proteome of different blood cell types, the selection of k=4 initial clusters resulted in clearly distinguishable trends that are shown as centroid profile diagrams for either dataset resulting in 16 trend intersections (the maximum possible for k=4) (Fig 3). Similarly, Hoogendijk *et al.* (2019) extracted 12 *modules* from the data, which represent combinations of trends in the transcriptome and proteome. The authors combined the modules based on their main trajectories, such as concordant increasing, concordant decreasing and increasing in the transcriptome while decreasing in the proteome. They classified the combined modules based on GO enrichment and the enrichment of specific database entries.

We visually identified four combined modules using OmicsTIDE by hovering the single links in the Sankey diagram (Fig. 3). As a next step we applied GO-enrichment analysis using OmicsTIDE to confirm the classifications. Fitting GO-terms were found for ‘RNA-binding protein’ (GO:0003723, RNA-binding Fig. 3a) and ‘Granule Development’ (GO:0042581, specific granule, Fig. 3b). For ‘biosynthesis and metabolism’ (Fig. 3c) we found a number of terms that are involved in these processes (e.g. GO:1901566, GO:0009205, GO:0006754) rather than finding a single fitting term. A reason for this could be that ‘biosynthesis and metabolism’ is a very broad category involving a lot of GO-terms.

The annotation ‘ROS machinery’ could not be solely reproduced using the GO-enrichment of OmicsTIDE (Fig. 3d), since the authors used a combination of GO enrichment and manual enrichment using other databases specialized on the annotation of human proteins. To confirm that the underlying gene sets are similar we compared them manually. Since the authors grouped the modules into broader categories the sets of concordant genes stemming from both decreasing trends were merged for OmicsTIDE as well. Overall, OmicsTIDE produced 617 increasing concordant genes, while 621 were found in the blood cell study with an overlap of 486 genes (Supplementary Table S1). This indicates that OmicsTIDE includes a trend intersection containing a gene set similar to the one annotated with ‘ROS machinery’. Similarly, of the 1320 decreasing concordant genes, 1131 could be found in similar patterns in OmicsTIDE (yellow and green trend, total of 1439 genes). The other modules compared were much smaller and we found more genes in OmicsTIDE. Yet, we could find more than 70% of the genes of each module.

### 5.2 Transcriptome and proteome time series data set of *Streptomyces coelicolor*

To demonstrate how *inter-omics* as well as *intra-omics* analysis can be combined using OmicsTIDE, we re-analyzed the datasets of a study exploring two *Streptomyces coelicolor* strains with respect to changes in their metabolisms under phosphate-starving growth conditions in a time-course experiment (Sulheim *et al.*, 2020). The *Streptomyces coelicolor* strains M145 and M1152 were used to study the role of *biosynthetic gene clusters* (BGCs) for the production of antibiotics. M1152 is a genetically engineered derivate of the M145 wild-type strain that was subject to the deletion of different BGCs (Gomez-Escribano and Bibb, 2011). For both strains samples were collected at eight timepoints. Phosphate was depleted between timepoint 3 and timepoint 4. For each of the time points, three biological replicates were generated for each omics layer. Both, transcriptome and proteome data was first quantified and log_2_-transformed. Next, the data was normalized by an *intra-strain* and *intra-omics* quantile-normalization across all replicates. Finally, the mean of the three replicates was calculated.

In OmicsTIDE the four datasets (M145 transcriptome, M1152 transcriptome, M145 proteome, M1152 proteome) were loaded resulting in six pairwise trend comparisons. For the *k*-Means clustering k=4 was chosen since it produced the most clearly distinguishable trends. We first focused on the comparison of two different strains across a single omics layer (M1152 transcriptome versus M145 transcriptome) to find differences on the transcript level. The insights from this first pairwise comparison were then used to study whether these insights are reflected in the proteome of the mutant strain. In particular this inter-omics comparison had not been subject to the study of Sulheim *et al.* (2020).

#### 5.2.1 Intra-omics: M1152 transcriptome versus M145 transcriptome

**Fig. 4.**
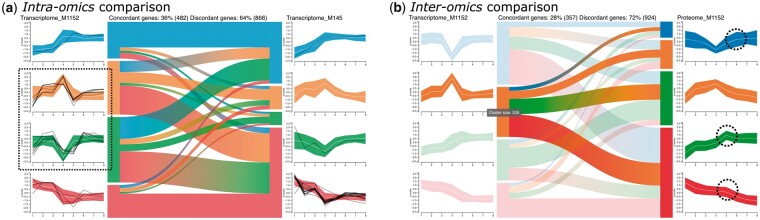
Combination of *intra-omics* and *inter-omics* analysis for *Streptomyces coelicolor*. (**a**) First-level analysis of the transcriptomes of the strains M1152 and M145 after focusing on genes with a median abundance above the 80th percentile. A custom list containing genes involved in the metabolic switch to nitrate respiration under phosphate starvation has been highlighted (black lines). The green and orange trend show exactly inverse patterns. (**b**) First-level analysis of M1152 transcriptome (left) versus M1152 proteome (right) trend in the transcriptome. Only a small fraction of genes follows the same trend in the proteome. Three trends in the proteome have a peak at a later timepoint than the peak of the orange trend in the transcriptome

The *intra-omics* comparison of the M1152 transcriptome and the M145 transcriptome revealed a total of 7904 genes that appear in both datasets, whereof around 55% follow concordant trends (data not shown). After applying the abundance filtering to focus on genes with a high median abundance of above the 80th percentile in both datasets the shape of the trends becomes clearly visible (Fig. 4a). Interestingly, the centroid profile plots show that the green trend and the orange one show the exact inverse trend in the M1152 transcriptome. The same can be observed for the blue trend and the red trend. The inverse behavior of the trends is also partly reflected in the M145 transcriptome. However, about 64% of the genes show discordant expression trends, indicating that the effect of phosphate depletion on gene expression differs between the two strains.

These findings were investigated in more detail by combining the trend comparison with information on genes involved in the metabolic switch to nitrate respiration under phosphate depletion (Martín *et al.*, 2017). The corresponding gene IDs were highlighted in the profile diagrams (Fig. 4a). Intriguingly, all but one highlighted gene follow the same trend in the M145 transcriptome and are downregulated until timepoint four (red trend), followed by an up-regulation. In the M1152 transcriptome the genes are distributed across three trends, most strikingly the green and orange trend where the highlighted genes show a peak at time point 4, but go back to the expression level observed before the depletion.

#### 5.2.2 Inter-omics: M1152 transcriptome versus M1152 proteome

We investigated how the patterns of trends co-occurring with phosphate depletion in the M1152 transcriptome are reflected in the corresponding proteome (Fig. 4b, Supplementary Fig. S3). The peaks in the blue and orange trends of the transcriptome appear shortly after phosphate depletion and the trends show low concordance with trends in the proteome. In contrast, the green and red trends show constantly increasing and decreasing behaviors with high concordance in the proteome. Therefore, we can conclude that the transcriptomic trends with peaks associated with phosphate depletion are not directly evident in the proteome, while the constant trends are more concordant in their behavior across the two omics layers. However, when hovering over the orange trend, we detected that the remaining three trends in the proteome all share a small peak at a later time point (Fig. 4b). Since this small peak appears at a later time point than the peak in the orange trend of the M1152 transcriptome, it could be further investigated whether this suggests a time-delayed translation of the protein cognates.

## 6 Discussion

In this article, we present OmicsTIDE, an easy to use analysis and visualization tool for the concurrent exploration of multi-omics data implementing our goal of interpretability (Goal 1).

In the context of developing OmicsTIDE we also devised a classification system for multi-omics data, which offers an underlying framework for our tool, but may also serve useful for future developments in this field. The interactive trend comparison in OmicsTIDE using the concept of concordance and discordance emphasizes the similarities and differences between two omics datasets. With this, it marks an innovation compared to other tools that mainly aim to integrate a large number of omics datasets to derive a combined pattern. It should be noted that the pairwise analysis and the multi-omics integration are not mutually exclusive ways of analysis, but rather complement each other.

OmicsTIDE uses a Sankey diagram to compare trends across datasets. With this visualization, concordance and discordance between trends can be intuitively explored. The trends are either visualized using *centroid profile plots*, profile plots or boxplots. While centroid profile plots visualize an overview of the profile, detailed profile plots show every gene separately. With this detailed visualization it is easier to track the behaviour of single genes. Profile plots are especially useful if the order of conditions is inherent, such as time series (Gehlenborg and Wong, 2012). In contrast, boxplots do not assume that the conditions are ordered and are, therefore, better suited for categorical data. Moreover, they focus on visualizing the distribution of values at each condition. This is especially useful to identify outliers or for assessing consistency across replicates.

To compute trends from multi-omics data, OmicsTIDE uses an *early integration* approach by first concatenating and then clustering the data. Currently, for the clustering k-Means++ is applied in OmicsTIDE. In addition, OmicsTIDE can use any early integration clustering uploaded manually by the user. While we were able to show that applying k-means extracts the main trends which can clearly be distinguished, we plan to implement more sophisticated clustering algorithms, such as dbscan (Ester *et al.*, 1996) or iCluster (Shen *et al.*, 2009) in a future version. Such approaches might prevent biased trends, especially if the number of genes in one of the compared datasets is very high compared to the other dataset. To counteract this bias, we analyze intersecting and non-intersecting genes separately, which guarantees an equal number of genes for both datasets in the intersecting analysis.

The ability of OmicsTIDE to extract and compare trends was demonstrated in two case studies using different experimental designs. In the first case study, the integrated analysis of transcriptome and proteome data shows that OmicsTIDE can derive the most important information in few steps leading to findings similar to the ones in the original study. These findings were further consolidated by a manual comparison of the genes extracted from the intersections of the trends in OmicsTIDE and the modules defined by the authors of the publication. Although the modules could not be reproduced perfectly in OmicsTIDE due to the much simpler clustering approach, between 70% and 85% of the genes found in the respective modules agreed with the trends identified in OmicsTIDE.

The second case study applies a more complex experimental design enabling an *intra-omics* as well as *inter-omics* comparison. As OmicsTIDE provides the option of combining different pairwise omics data comparisons within a single analysis according to our third goal, trends could be analyzed in the *intra-omics* as well as the *inter-omics* comparison while keeping an overview of all involved datasets. The exploration of the Sankey diagram using the *focus-on-hover* strategy could show that the trends initially found in the intra-omics analysis (the transcriptome comparison) are also revealed in the proteome. In summary, the parallel analysis of *intra-* and *inter-omics* data in OmicsTIDE leads to easily interpretable expression trends and possible hypotheses.

In OmicsTIDE, we compare datasets using shared keys (e.g. gene IDs), which facilitates the comparative visualization of trends. In a future version, a pairwise comparison between omics layers not sharing keys and an advanced comparison of non-intersecting genes could be achieved by linking keys using meta-information, such as common pathway IDs.

With OmicsTIDE we present a tool for initial exploration and hypothesis generation, which complements advanced statistical or machine-learning methods. The choice of additional analysis methods depends on the generated hypothesis. Yet, in future versions, we plan to integrate methods for statistical validation of the extracted trends.

OmicsTIDE is designed in particular for biologists; its user interface creates clear default views that show the concordant and discordant patterns in omics abundance data in a pairwise manner. The simple input format (numerical matrices) leads to great flexibility in OmicsTIDE as it can perform *inter-omics* as well as *intra-omics* comparisons, thus allowing for example also the comparison of two transcriptomic, proteomic or metabolomic datasets as well as the analysis of complex mixed-omics experimental designs.

## Supplementary Material

vbac093_Supplementary_Data

## Data Availability

The data underlying this article are available in the article and in its online supplementary material.
