# Supplementary material for: OmicsTIDE: interactive exploration of trends in multi-omics data
Source: Bioinform Adv. 2023 Jan 20;3(1):vbac093. doi: 10.1093/bioadv/vbac093 (PMC9869718; doi:10.1093/bioadv/vbac093)
Supplement: vbac093_Supplementary_Data [file vbac093_supplementary_data.pdf]

Supplementary Material

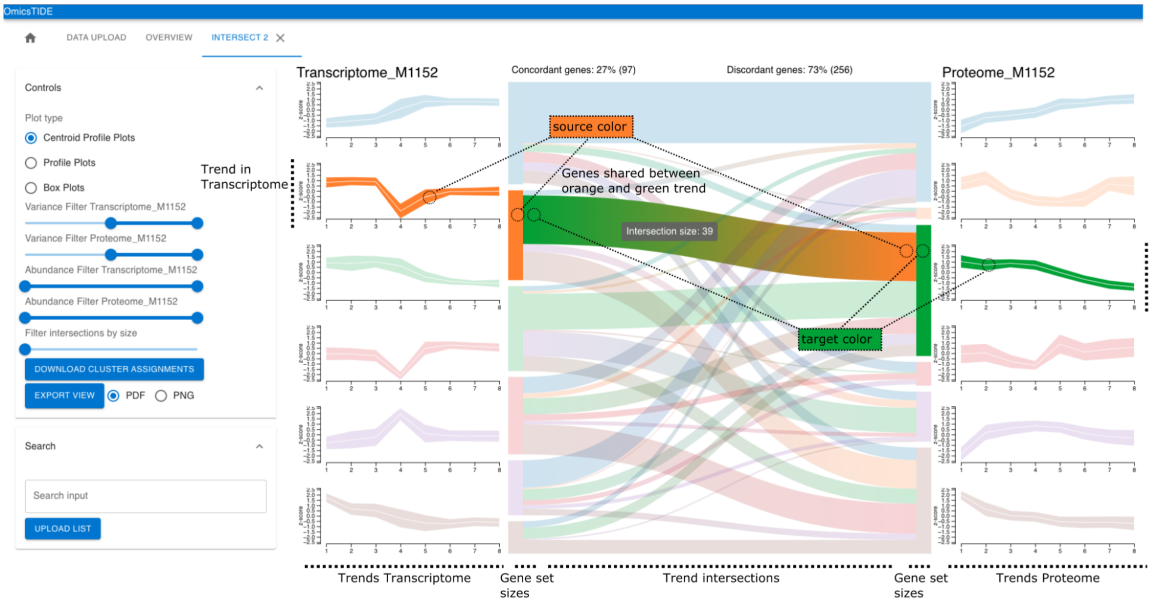

Fig. S1: Screenshot of OmicsTIDE. OmicsTIDE employs a tab-based design for easy exploration of multiple comparisons. The selected tab (Intersect 2) corresponds to the intersecting analysis of the second comparison. The visualization consists of centroid profile plots representing trends of genes as well as a Sankey diagram showing the intersections of trends between the data sets. Each trend represents a set of genes belonging to a cluster and a data set. The trends are visualized as centroid profile plots and colored with a set of categorical colors. These colors are repeated in the nodes of the Sankey diagram showing the sizes of the gene sets corresponding to the trends. The bands (links) of the Sankey diagram encode for the intersections between the trends. The band connecting the orange and green trend has been highlighted. The color gradient of the bands is inverted to allow a quick identification of all target nodes belonging to a source node. In the sidebar (on the left), interactive controls allow the user to change plot types of the trends, filter the data, export results, and search for genes.

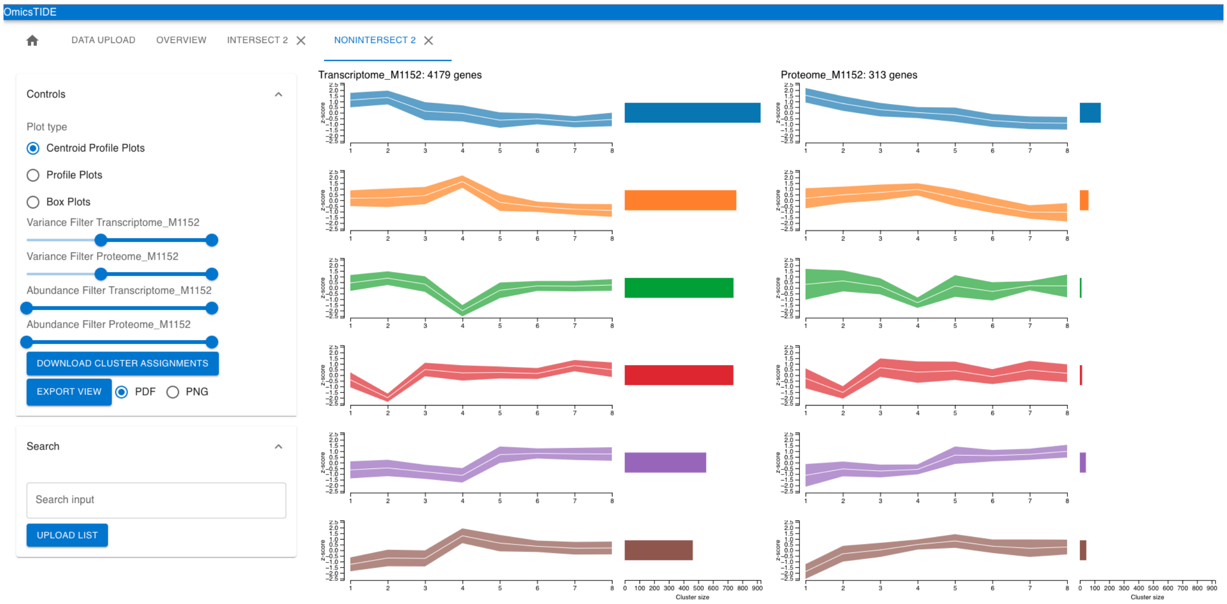

Fig. S2: First level analysis of non-intersecting genes. For non-intersecting genes, trends can be visualized as centroid profile plots (as shown here), profile plots, and box plots. Gene set sizes are shown as horizontal bar charts, next to the respective profile plots.

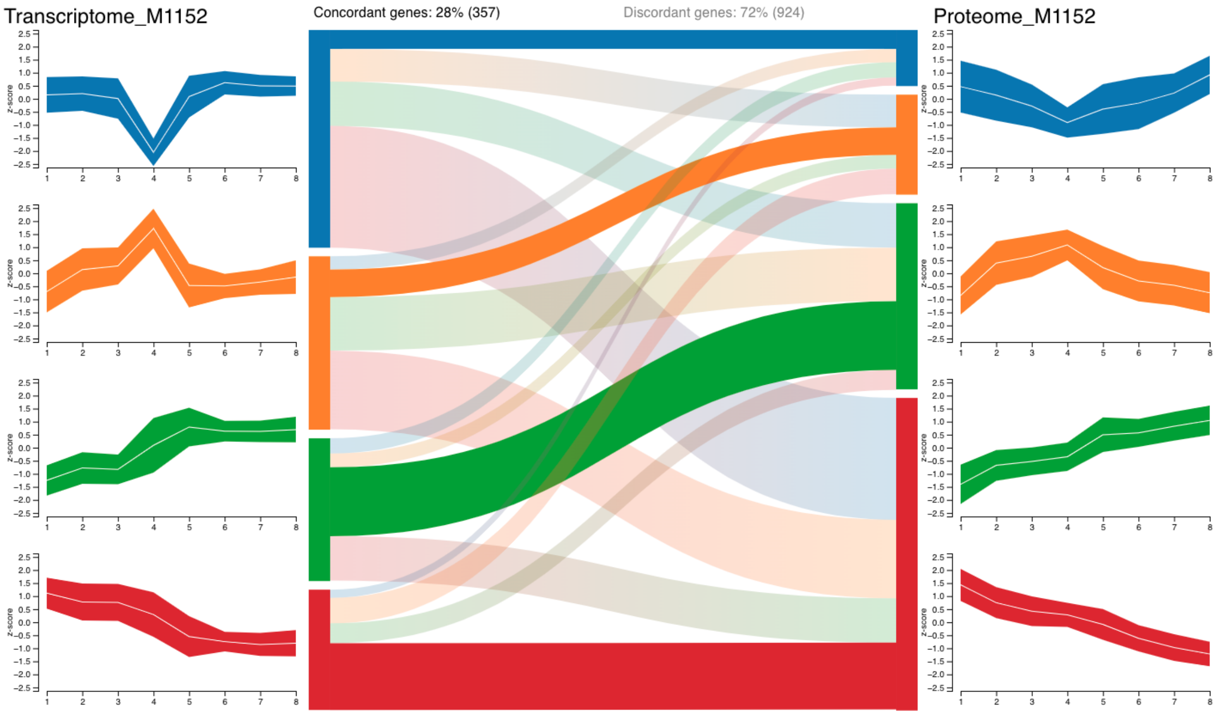

Fig. S3: The *inter-omics* trend comparison between the transcriptome and proteome of *S. coelicolor* M1152. The concordant intersections are highlighted. While the trends with prominent peaks (blue and orange) of the transcriptome show little concordance with the Proteome, the more constantly increasing (green) or decreasing (red) trends show higher *inter-omics* concordance.

Table S1: Comparison of modules created by Hoogendijk et al. with trend intersections created in OmicsTIDE.

Trend transcriptome	Increasing	Decreasing	Increasing	Curved
Trend proteome	Increasing	Decreasing	Decreasing	Increasing
Function (Hoogendijk et al.)	Ros Machinery	Biosynthesis and metabolism	RNA-binding proteins	Granule development
# genes (Hoogendijk et al.)	621	1320	212	192
# genes OmicsTIDE	617	1439	508	270
Intersection	486	1131	177	132

Supplementary Video Case Study 1: https://www.youtube.com/watch?v=LACWizg26hE
Supplementary Video Case Study 2: https://www.youtube.com/watch?v=bvIoSnKTjWA
